# NARFL deficiency caused mitochondrial dysfunction in lung cancer cells by HIF-1α–DNMT1 axis

**DOI:** 10.1038/s41598-023-44418-7

**Published:** 2023-10-11

**Authors:** Hongzhou Liu, Xueqin Wu, Tianrong Yang, Chen Wang, Fei Huang, Ying Xu, Jie Peng

**Affiliations:** 1 School of Clinical Medicine, The First Affiliated Hospital of Chengdu Medical College, 783# Xindu Avenue, Chengdu, 610500 Sichuan Province People’s Republic of China; 2 Department of Medical Laboratory, The Central Hospital of Wuhan, Tongji Medical College, Huazhong University of Science and Technology, 26# Shengli Road, Wuhan, 430014 Hubei Province People’s Republic of China; 3 Department of Clinical Laboratory, The Third People’s Hospital of Chengdu, 82# Qinglong Street, Chengdu, 610014 Sichuan Province People’s Republic of China; 4 Center of Clinical Laboratory, The First Affiliated Hospital of Soochow University, Suzhou, 215006 Jiangsu Province People’s Republic of China

**Keywords:** Cancer genetics, Cancer metabolism, Cancer prevention, Cancer therapy, Lung cancer

## Abstract

NARFL was reported to be a component of cytosolic iron–sulfur cluster assembly pathway and a causative gene of the diffused pulmonary arteriovenous malformations (dPAVMs). NARFL knockout dramatically impaired mitochondrial integrity in mice, which might promote mitochondrial dysfunction and lead to worse survival rate of lung cancer. However, the underlying molecular mechanism of NARFL deficiency in non-small cell lung cancer (NSCLC) is unknown. Knockdown assay was performed in A549 and H1299 cells. The protein levels of HIF-1α and DNMT1 were measured, and then Complex I activity, mtDNA copy numbers and mRNA levels of mtND genes were determined. Cisplatin resistance and cell proliferation were conducted using CCK8 assay. Cell migration and invasion were detected using wound heal assay and transwell assay. Survival analysis of lung cancer patients and KM plotter database were used for evaluating the potential value of NARFL deficiency. NARFL protein was expressed in two cell lines and knockdown assay significantly reduced its levels. Knockdown NARFL increased the protein levels of HIF-1α and DNMT1, and downregulated the mRNA levels of ND genes, mitochondrial Complex I activity, mtDNA copy number, and ATP levels. The mitochondrial dysfunction caused by NARFL deficiency were ameliorated by siHIF-1α and DNMT1 inhibitor. Knockdown NARFL increased the drug resistance and cell migration, and siHIF-1α reversed this effect. Moreover, NSCLC patients with NARFL deficiency had a poor survival rate using a tissue array and KM plotter database, and it would be a target for cancer prognosis and treatment. NARFL deficiency caused dysregulation of energy metabolism in lung cancer cells via HIF-1α–DNMT1 axis, which promoted drug resistance and cell migration. It provided a potential target for treatment and prognosis of lung cancer.

## Introduction

Lung cancer is a malignant tumour originating from bronchial epithelial cells and is the main cause of cancer-related death in China^1^. More than 80% of them are non-small cell lung cancer (NSCLC) based on morphological characteristics. Surgery, drug therapy and radiotherapy are adopted for treatment of NSCLC^2^. However, the prognosis of some patients is still poor due to tumour progression, and their 5-year survival rate is very low. Some mechanisms of tumour progression have been elucidated in terms of genes and signaling pathways involved in mitochondrial dysfunction^3,4^, while the underlying issues still need further research.

The mitochondria are the powerhouse of the cell, and play a central role in cellular oxidative phosphorylation, which are consisted of complex I, complex II, complex III, complex IV or cytochrome c oxidase (CO), and complex V^5,6^. Mitochondria compose of multiple copies of mitochondrial DNA (mtDNA), which is a 16.6-kb double-stranded DNA and encodes 13 complex polypeptides (such as NADH dehydrogenase, ND1-ND6 and ND4L), 22 tRNAs, and 2 rRNAs^6^. Decreased mtDNA copy number and dysregulation of ND genes are expected to affect energy production, cell migration and cancer survival^6^. It was shown that depletion of mtDNA impaired mitochondrial Complex I activity and resulted in resistance to Adriamycin in Hela cells^7^. Depletion of mtDNA enhanced the expression of the multidrug resistance 1 (MDR1) gene, which would drive tolerance to anti-cancer drugs in cancer cells^8–10^.

Human cancers can endure profound hypoxia conditions, which facilitate the mitochondrial dysfunction, and adaptation to it is also an important step in tumorigenesis. The activation of HIF-1α is a critical adaptive regulator for hypoxia, which is transported into the nucleus, and forms a heterodimer with HIF-1β, and then binds to the hypoxia-responsive element in the promoter regions of glycolytic enzymes, such as GLUT1 and LDHA^6^. It could also promote the expression of PDK1, which inhibits the conversion of pyruvate to acetyl-CoA and represses the mitochondrial oxidative function^11^. Activation of HIF-1α under normoxic/hypoxic conditions is an important feature in cancer cells, which led to metabolic reprogramming (such as Warburg effect), and upregulation of downstream genes, including DNMT1^12,13^. This malignant phenotype of cancer cells increases glucose absorption and methylations of tumor suppressors, which provides enough energy for proliferation and drives invasion and progression^12^. Abnormal activation of HIF-1α caused by nuclear prelamin A recognition factor-like (NARFL) deficiency has been illustrated^14–17^. However, the mechanism of NARFL deficiency involved in mitochondrial dysfunction and tumour progression remains to be elucidated.

NARFL, also known as CIAO3, is a component of cytosolic iron–sulfur cluster (Fe–S) assembly pathway in human cells^14^. In previous studies, knockdown NARFL led to impaired activity of cytosolic aconitase and increased levels of HIF-1α protein under normoxic/hypoxic conditions in mammal cells^15^. In Zebrafish, narfl deletion also increased HIF-1α activation through reactive oxygen species (ROS), which resulted in subintestinal vessel (SIV) malformation and digestive organ defects^16,17^. Moreover, knockout Narfl caused embryonic death and impaired mitochondrial integrity in mice^18^. These results suggested that NARFL deficiency could lead to upregulation of HIF-1α and cause mitochondrial dysfunction. Furthermore, in our previous work, NARFL (Ser161Ile) mutation was identified as a causative gene in diffuse pulmonary arteriovenous malformations (dPAVMs), and NARFL knockdown led to ROS overproduction by upregulation of NOX2 and NOX4 in endothelial cells^17,19^. However, the effect of NARFL deficiency in lung cancer cells need to be further researched.

## Materials and methods

### Patients and ethics statement

102 lung cancer tissues of NSCLC patients were collected from January 2007 to January 2012, and then they were fixed with 10% neutral buffered formalin for 24 h and embedded with paraffin. This study was approved by the Ethics and Scientific Committees of the First affiliated Hospital of Chengdu Medical College, and complied with the Declaration of Helsinki, and written informed consents were obtained from all NSCLC patients. Some clinical data, such as gene mutations, adjuvant therapy before or after surgery were not fully collected, and it was hard to evaluate.

### QDs-IHC and overall survival analysis

The expression of NARFL was assessed using QDs-IHC staining (Jiayang Quantum Dots Co., Ltd. Wuhan). Briefly, the cancer tissues were prepared in graded alcohol, and then antigen retrieval was performed. NARFL antibody was diluted 1:200 tris-buffered saline and incubated with lung tissues. Goat secondary antibody was then incubated and QDs (605 nm) conjugated to streptavidin (1:400; Wuhan Jiayang) was used for staining. The signals of QDs-IHC were measured at 605 nm using Olympus BX53 fluorescence microscopy. All patients were divided into two groups based on the expression of NARFL. Overall survival (OS) of two groups was calculated using Kaplan–Meier method, which was the period from the date of initial diagnosis to death or the last follow-up. At the end of the study, 44 patients were still alive and 58 patients died of NSCLC. K–M plotter database was also selected for prognosis analysis.

### Cell culture

NSCLC cell lines A549 and H1299 were purchased from Procell company (Wuhan, China). Cells were cultured in RPMI-1640 medium (Hyclone, USA) with 10% fetal bovine serum (Gibco, USA) and 1% penicillin/streptomycin (Hyclone, USA) in humidified incubator at 37 °C and 5% CO_2_. Two cell lines were authenticated using Short tandem repeats (STR) profiling in 2022 (Wuhan, China). The results for STR were as follows: A549, AM: X,Y; D13S317: 11,11; VWA: 14,14; D16S539: 11,12; TH01: 8,9.3; CSF1PO: 10,12; D5S818: 11,11; D7S820: 8,11; TPOX: 8,11; and H1299, AM: X,X; D13S317: 12,12; CSF1PO: 12,12; D7S820: 10,10; VWA: 16,17,18; D16S539: 12,13; D5S818: 11,11; TH01: 6,9.3; TPOX: 8,8. Cells were treated with cisplatin (0.25 μM) for drug resistance in 96-well plates. And then cell proliferation was measured using CCK8 assay. Proliferation curves were plotted using absorbance values at each time point, which was read at 490 nm on a microplate reader. Wound healing assay and transwell assay were selected for assessment of cell migration.

### Overexpression or knockdown assay

pSUPER-retro-NARFL (shNARFL) plasmid was constructed for knockdown assay and pCMV-HA-NARFL plasmid was used for overexpression assay as previously described^17,19^. The empty vector and negative vector with a scramble oligo were used as the control plasmid. Two cell lines were transfected with plasmids for 24 h using Lipo3000 according to the manufacturer’s instruction. Stable cells of knockdown assay were selected in RPMI-1640 medium with 3 µg/ml blasticidin and puromycin for a week, and then the medium was replaced with RPMI-1640 containing 10% fetal bovine serum. qRT-PCR and Western blot were used for identifying the efficiency of knockdown and overexpression assay.

### Real-time qPCR

Total RNA from the cell lines was extracted using a RNeasy Mini Kit (Qiagen), and then genomic DNA was digested with DNase according to the manufacturer's instructions. The RNA concentration was measured using a micro ultraviolet–visible spectrophotometer (nanodrop, Thermo Fisher Scientifc), and 2000 ng of total RNA was reverse transcribed using a high-capacity reverse transcription Kit (Thermo Fisher Scientific) with random hexamers according to the manufacturer’s protocol. The relative expressions of genes were determined using SYBR Green PCR master mix (Takara) according to the manufacturer’s instructions. All data were acquired and analyzed on a Real-Time PCR Systems (Applied Biosystems) and the relative mRNA expression levels were calculated using the 2‑ΔΔ^Ct^ method.

### Complex activity measurement

Complex activities were measured using the complex assay kit (Abcam, ab109721 for Complex I activity, ab109905 for Complex II + III, ab109911 for Complex IV and ab109714 for Complex V) in mitochondrial fractions. A549 and H1299 cells were treated with NARFL shRNA in 6-well plates, and then they were lysed for protein collection according to the manufacturer’s protocol. Protein concentration was measured by BCA assay and a standard curve was quantified to ensure equal loading and activity measurement (Thermo Fisher Scientific). 25 μg protein of each sample from whole-cell lysate was using for complex I, IV and V activity, and 70 μg protein was used for complex II + III activity. Absorbance was measured spectrophotometrically and the results was performed triple times.

### mtDNA copy number assay

Total DNA from A549 and H1299 cell lines were extracted with DNeasy kit (Qiagen), and then it was diluted to 10 ng/μl. The relative levels of mtDNA were quantified using the RT-qPCR method. MT-ND4 gene and β-globin gene were obtain from NCBI database and primers were synthesized for PCR using SYBR mix. MT-ND4 gene refers to mtDNA sequence and β-globin is the nuclear DNA. All data were acquired and analyzed on a Real-Time PCR Systems (Applied Biosystems) using the 2^−ΔΔCt^ quantitative method.

### Transmission electron microscopy

Cell samples (1 mm × 1 mm × 2 mm) were quickly harvested and fixed with Gluta fixative (0.5%, 4 °C, Solarbio) for 10 min, and re-fixed with Gluta fixative (3%, 4 °C, Solarbio) for 2 h. The post-fixed cell samples were embedded, cut, and mounted at the Electron Microscopy Core Facility (Lilai Biotech), and then were analyzed using a Hitachi H-7500 transmission electron microscope (Hitachi, Tokyo, Japan) for mitochondria counting.

### Mitochondrial ATP measurement

ATP level was measured using an ATP assay kit (Beyotime). Briefly, the cell lysate of A549 and H1299 cells transfected with shRNA was centrifuged at 12,000 g for 10 min. 50 μl supernatant of cell lysate was collected and added into 100 μl ATP detection working solution. And then, luminescence was detected with Centro LB960 microplate luminescence detector (Berthold, Germany). The level of ATP was calculated according to the standard curve.

### Immunoblotting

A549 and H1299 cells were plated in 6-well plates and stably knockdown, and then total cells were collected by centrifugation at 3000×*g* for 5 min. It was washed using PBS buffer and lysed with RIPA lysis buffer (Beyotime, Beijing) according to the manufacturer’s instruction. 1 × protease cocktail and 10 mM PMSF (Sigma-Aldrich) were added for protease inhibition. The cell lysates were loaded on an SDS-PAGE gel, and then transferred to polyvinylidene difluoride (PVDF) membrane (Millipore Corporation). After blocked with skimmed milk, the membranes were incubated with primary antibodies against NARFL, HIF-1α, and DNMT1 (1:1000, all from Proteintech Shanghai, China), and GAPDH (1:3000, Beyotime Biotechnology) at 4 °C overnight. Following incubation with secondary antibodies (Beyotime Biotechnology), the blots were measured with an ECL kit (Beyotime Biotechnology) and the results were analyzed with image J software. All blots cut prior to hybridization with antibodies.

### Immunofluorescence (IF) assay

The stable knockdown cells were seeded in 24-well plates overnight and washed with precooled PBS for 10 min. And then they were fixed with 4% paraformaldehyde and permeabilized with 0.5% Triton X-100 at room temperature for 15 min. It was incubated with DNMT1 primary antibodies (1:500, Proteintech) at 4 °C overnight, and then washed with PBS and incubated with fluorescent secondary antibody at room temperature for 2 h. DAPI dihydrochloride (Beyotime) was used to label the nuclei. The results were analyzed using the ImageJ software.

### Statistical analysis

SPSS17.0 and Prism 5.0 were used for statistical analysis. Cell experiments were performed at least 3 independent replicates and the results were presented as mean ± SD. Student’s t-test was used to determine the statistical significance (Prism 5.0). ANOVA and the S–N–K test, such as a post-hoc test, were used for comparisons of multiple groups. Survival analysis was detected by Kaplan–Meier method and log-rank test. K–M plotter database was also selected for survival analysis. Statistically significant differences were considered when two-tailed *P*-values < 0.05 was considered as statistically significant.

## Results

### Knockdown NARFL caused mitochondrial dysfunction in two cell lines.

**Figure 1:**
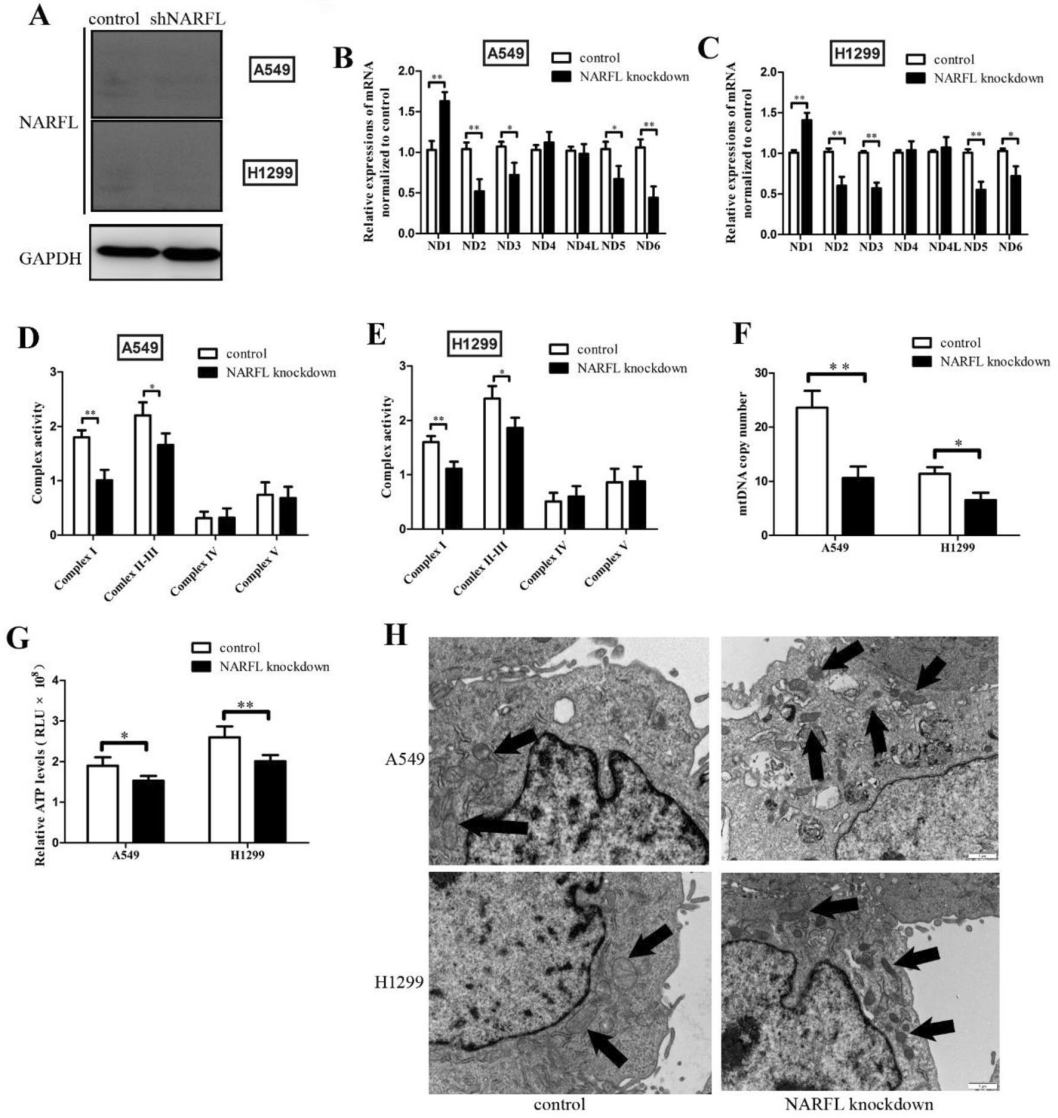
Knockdown NARFL caused mitochondrial dysfunction in two cell lines. (**A**) Knockdown assay was performed using shRNA, and the results showed that it was effective for NARFL knockdown in A549 and H1299 cells. (**B**–**C**) mtND1-6 and mtND4L mRNA levels were detected using RT-qRCR method. ND2, ND3, ND5 and ND6 was significantly downregulated in A549 cells (**B**) and H1299 cells (**C**), but increased in ND1. (**D**–**E**), Mitochondrial Complex activities were measured using activity kit, and NARFL knockdown decreased the activities of Complex I and Complex II–III, but not Complex IV and Complex V. (**F**) mtDNA copy number was tested and it was significantly downregulated in two cell lines treated with NARFL knockdown. (**G**) ATP levels were significantly reduced in two cells using NARFL knockdown. These results indicated that NARFL knockdown could affect the ND genes expressions and mitochondrial Complex activities, and it would downregulate the mitochondrial complex activity and energy generation. (**H**) TEM was used for observation of mitochondria. Mitochondrial membrane density was increased, but the quantity was decreased in two cells.

As shown in Fig. 1A, knockdown assay was effective in two cell lines and protein levels of NARFL were significantly downregulated. The mRNA levels of ND2, ND3, ND5 and ND6 were decreased in NARFL knockdown cells (Fig. 1B–C). However, ND1 gene was dramatically increased. We next examined the activities of mitochondrial Complex I–V, and decreased activities of Complex I and Complex II–III were observed in Fig. 1D,E, but not in Complex IV and Complex V. These results indicated that NARFL knockdown downregulated the mRNA expressions of ND genes and the activity of Complex I, which might cause mitochondrial dysfunction. To assess the mitochondrial dysfunction, we next determined mtDNA copy number and ATP levels in knockdown cells. Knockdown NARFL was shown to decrease mtDNA copy number in two cell lines in Fig. 1F, and NARFL knockdown also reduced the ATP levels in H1299 cells (Fig. 1G). And subsequently, the results of transmission electron microscopy showed that mitochondrial membrane density was increased, and the quantity of mitochondria was decreased in Fig. 1H. This result indicated that inadequate NARFL downregulated the mitochondrial functions, and might drive the metabolic reprogramming.

### NARFL knockdown caused mitochondrial dysfunction via HIF-1α activation

**Figure 2:**
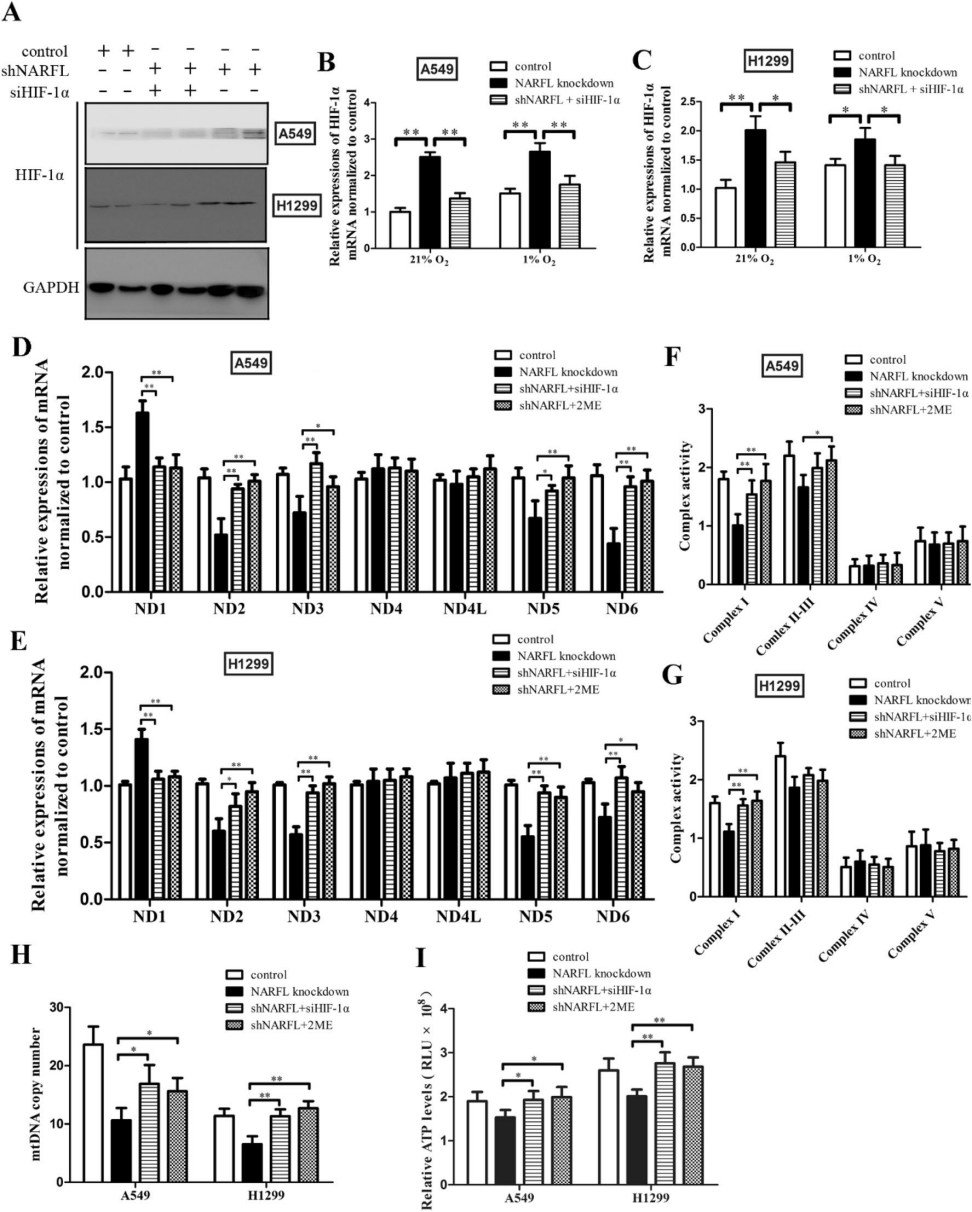
NARFL knockdown caused mitochondrial dysfunction via HIF-1α activation. (**A**) Knockdown NARFL increased HIF-1α expression and this effect was inhibited using siHIF-1α. (**B**–**C**) HIF-1α mRNA levels were also significantly increased in NARFL knockdown A549 (**B**) and H1299 (**C**) cells in normoxic/hypoxic conditions. (**D**–**E**) HIF-1α inhibition and 2ME2 treatment improved the effects of NARFL deficiency on ND genes in A549 (**D**) and H1299 (**E**) cells. F-I, HIF-1α inhibition and 2ME2 treatment also ameliorated the effects of NARFL deficiency on Complex I activity in A549 (**F**) and H1299 (**G**) cells, mtDNA copy number (**H**) and ATP levels (**I**). These results suggested that NARFL knockdown induced dysregulation of mitochondrial function via HIF-1α.

We next examined the expressions of HIF-1α to explore its underlying mechanism of mitochondrial dysfunction. Knockdown NARFL was shown to increase the protein levels of HIF-1α, and siRNA decreased the HIF-1α expressions in Fig. 2A. We then measured the mRNA levels of HIF-1α under 21% O_2_ and 1% O_2_ conditions. As shown in Fig. 2B–C, HIF-1α mRNA levels both increased under normoxic/hypoxic conditions in NARFL knockdown cells. siHIF-1α treatment reduced the HIF-1α mRNA levels and reversed the effects of NARFL deficiency on ND genes expressions (Fig. 2D–E), Complex I activity (Fig. 2F–G), mtDNA copy number (Fig. 2H), and ATP levels (Fig. 2I). 2-Methoxyestradiol (2ME2, HIF-1α inhibitor) treatment also improved the mitochondrial dysfunction caused by NARFL deficiency (Fig. 2D–I). These results indicated that NARFL deficiency induced mitochondrial dysfunction though activation of HIF-1α, and inhibition of HIF-1α reversed the effects of NARFL deficiency.

### NARFL insufficient upregulated the expression of DNMT1 through HIF-1α

**Figure 3:**
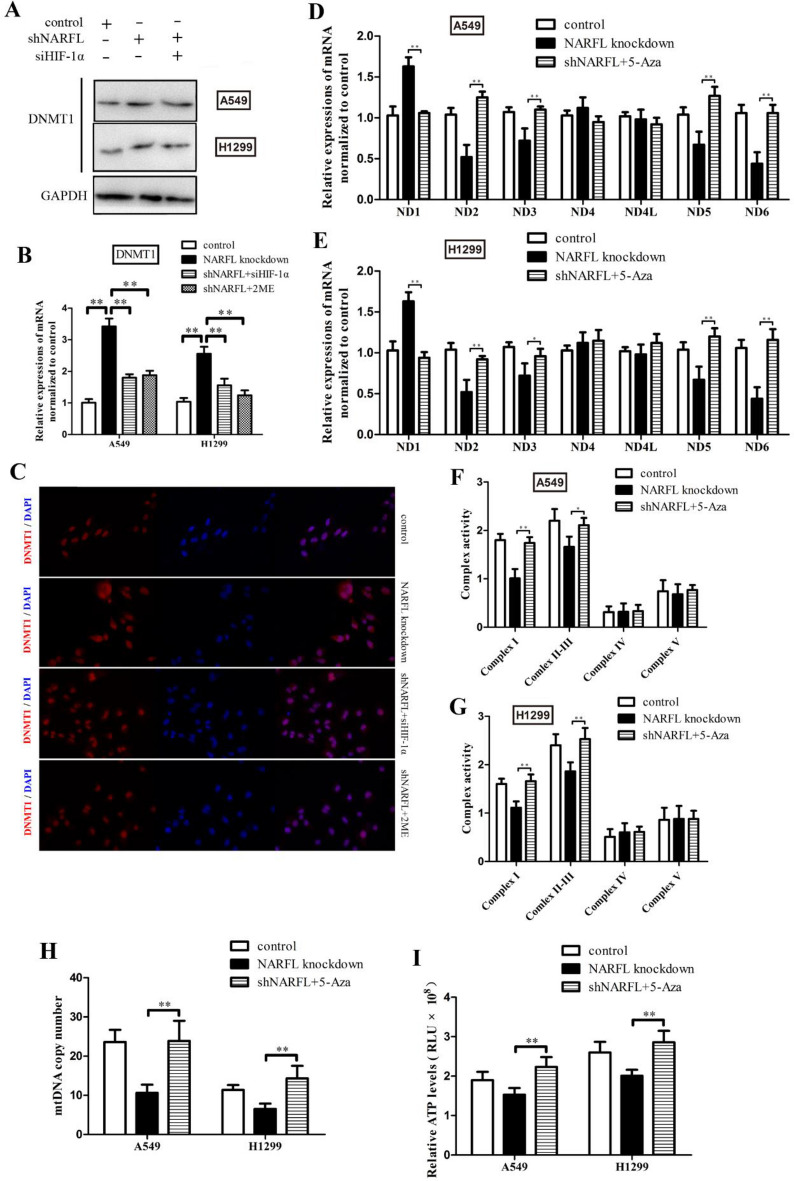
NARFL insufficient upregulated the expression of DNMT1 through HIF-1α. (**A**–**B**) Knockdown NARFL increased the protein levels (**A**) and mRNA levels (**B**) of DNMT1. siHIF-1α and 2ME treatment could reverse this effect. (**C**) The results of immunofluorescence also showed that knockdown NARFL increased the DNMT1 expression and this effect was eliminated by HIF-1α inhibition. (**D**–**E**) 5-Aza treatment improved the dysregulation of ND genes in A549 cells (**D**) and H1299 cells (**E**). (**F**–**I**) 5-Aza treatment also ameliorated the effects of NARFL deficiency on Complex I activity in A549 (**F**) and H1299 (**G**) cells, mtDNA copy number (**H**) and ATP levels (**I**).

As the downstream gene of HIF-1α, DNMT1 was also measured to explore the alteration of epigenetics. NARFL knockdown was shown to increase the expression of DNMT1 and siHIF-1α reduced the protein levels of DNMT1 in Fig. 3A. We then detected the mRNA levels of DNMT1, and they were also increased in the NARFL knockdown cells. Moreover, HIF-1α inhibition ameliorated the expressions of DNMT1 in Fig. 3B. The increased DNMT1 expressions were also determined using immunofluorescence in Fig. 3C, and HIF-1α inhibition could reverse the upregulation of DNMT1. These results proved that NARFL insufficient activated the HIF-1α–DNMT1 axis, and might affect mitochondrial gene expressions in terms of epigenetic mechanisms. Subsequently, 5-Azacytidine was chosen for DNMT1 inhibition, and the results showed that 5-Azacytidine treatment reversed the effects of NARFL deficiency and improved the mRNA levels of ND genes (Fig. 3D–E), Complex I activity (Fig. 3F–G), mtDNA copy number (Fig. 3H), and ATP levels (Fig. 3I). These results suggested that NARFL deficiency caused dysregulation of mtDNA due to epigenetic mechanisms, and activation of HIF-1α–DNMT1 axis was responsible for mitochondrial dysfunction and reduced energy production in NARFL knockdown cells.

### NARFL knockdown caused drug resistance and promoted cell migration

**Figure 4:**
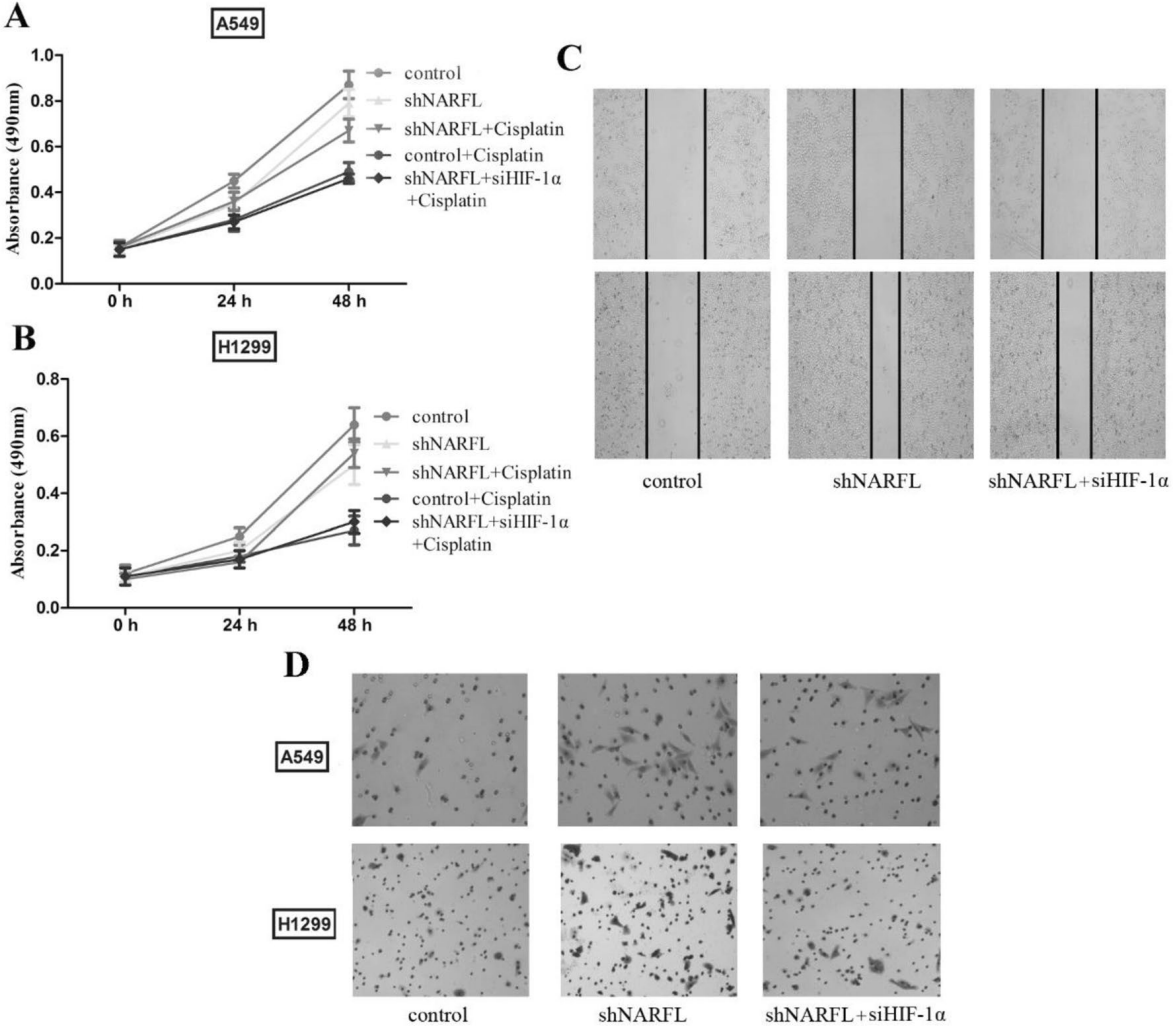
NARFL knockdown caused drug resistance and promoted cell migration. (**A**–**B**) Cell survival was tested using Cisplatin and proliferation curves were plotted in A549 cells (**A**) and H1299 cells (**B**). The results showed that NARFL knockdown tended to decrease the cell proliferation, and it was significantly reduced in two cell lines treated with Cisplatin. Inhibition of HIF-1α eliminated the chemotherapy drug resistance induced by NARFL deficiency. (**C**–**D**) NARFL deficiency promoted cell migration (**C**) and invasion (**D**) in A549 cells. siHIF-1α reversed its effects. These results indicated that NARFL knockdown caused drug resistance and cancer invasion via HIF-1α pathway.

The energy generation was affected by NARFL deficiency based on the above results, cisplatin resistance and cell migration were then measured for further evaluation. As shown in Fig. 4A–B, shNARFL tended to decrease the cell proliferation of two cell lines, while it was not significantly different. Cell proliferation was dramatically reduced in two cell lines treated with Cisplatin, and knockdown NARFL reversed the effect of Cisplatin. Inhibition of HIF-1α eliminated the chemotherapy drug resistance induced by NARFL deficiency. These results indicated that NARFL knockdown caused drug resistance via HIF-1α pathway and wound promote cell survival in lung cancer patients treated with Cisplatin. Cell migration was also increased as shown in Fig. 4C–D, and proved that NARFL deficiency promoted cell invasion. These results suggested that NARFL deficiency would promote drug resistance and cancer metastasis, which might lead to worse survival rate.

### NARFL deficiency predicted a poor survival rate

**Figure 5:**
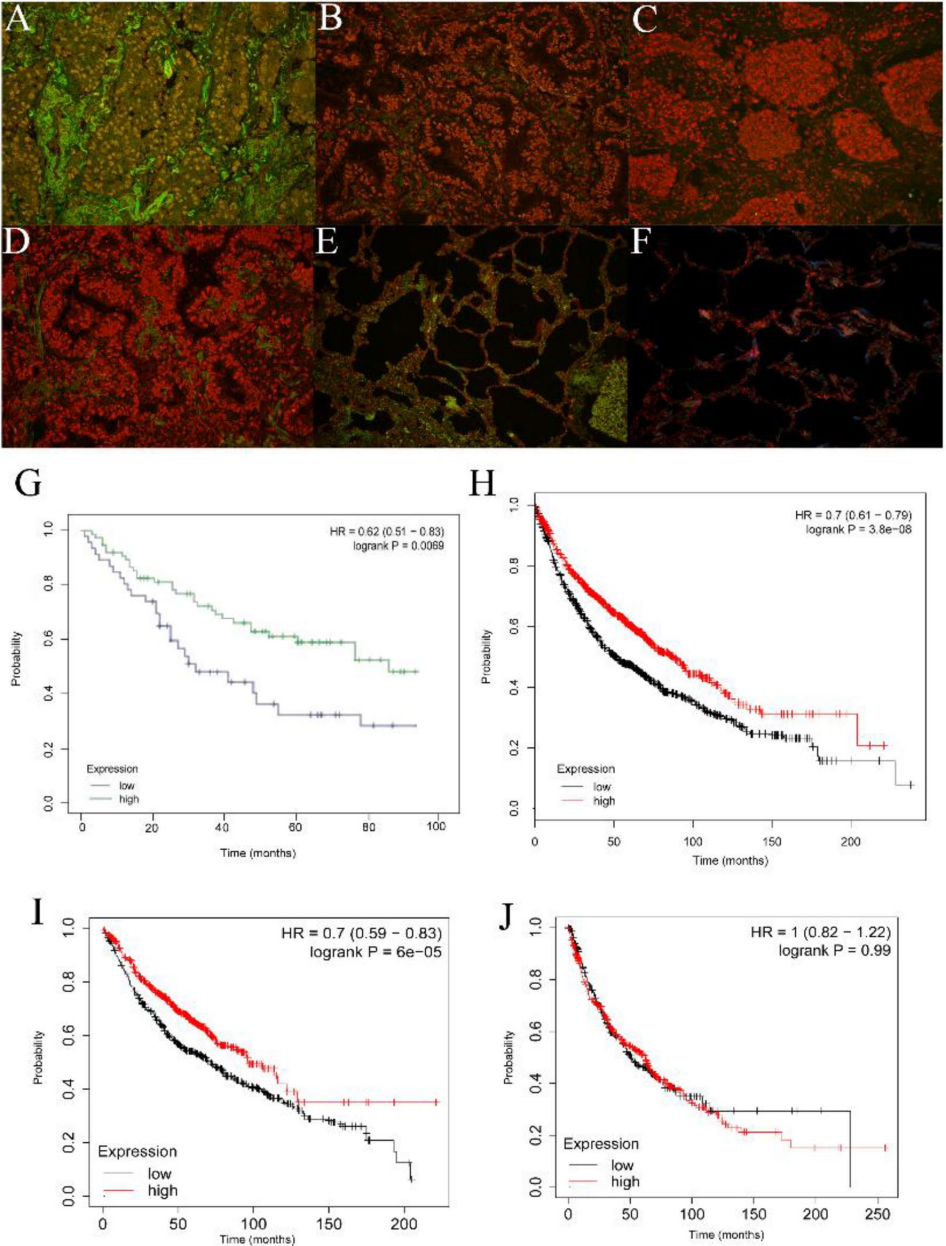
NARFL deficiency predicted a poor survival rate. A-D, Expressions of NARFL were detected using QDs-IHF method. 1 score of NARFL expression (**A**), 2 scores (**B**), 3 scores (**C**), and 4 scores (**D**) were showed and total scores was calculated using image J software. (**E**–**F**) NARFL expression was also measured in adjacent cancer tissues (**E**) and normal lung tissues (**F**). (**G**–**H**) Overall survival rate was measured using K–M test and NARFL low expression predicted a poor survival rate in 102 patients (**G**) and 1926 patients (**H**). (**I**–**J**) Overall survival rate was also detected in adenocarcinoma (**I**) and squamous cell carcinoma (**J**), and patients with low NARFL expression had a poor prognosis in adenocarcinoma, but not in squamous cell carcinoma.

Expressions of NARFL were next determined using QDs-IHF in lung cancer patients, and the results showed that NARFL expression levels were obviously different in NSCLC patients (Fig. 5A–F). The scores of QDs-IHF were calculated and all patients were divided into two groups based on the mean value. Patients with low expressions of NARFL had a poor survival rate as shown in Fig. 5G. The result of Fig. 5H was also proved that a poor survival rate was observed in 1926 NSCLC patients with low NARFL expressions. In Fig. 5I, low expression of NARFL also had a poor survival rate in 1308 patients with adenocarcinoma, but not in patients with squamous cell carcinoma (Fig. 5J). These results demonstrated that NARFL deficiency would be a predictor for treatment and prognosis of lung cancer, especially for adenocarcinoma.

## Discussion

We found that knockdown NARFL caused mitochondrial dysfunction via HIF-1α–DNMT1 axis, which resulted in dysregulation of ND genes and mtDNA copy number. Moreover, NARFL deficiency induced drug resistance and promoted cell migration, and overall survival analysis demonstrated that NARFL deficiency was a predictor for a poor prognosis.

Knockdown NARFL was reported to increase HIF-1α expression^14,15^, and our results presented that HIF-1α was also upregulated caused by NARFL knockdown in lung cancer cell lines. Upregulation of DNMT1 was also observed and translocated into nucleus as shown in Fig. 5C, which would drive methylation of nuclear genes, such as TK2 and TFAM. Methylation and downregulation of TK2 caused reduction of nucleotides required for mtDNA synthesis and reproduction, which led to mtDNA depletion and ATP levels reduction^13,20^. As shown in Fig. 2A, NARFL knockdown reduced mtDNA copy number, which proved the existence of mtDNA depletion and metabolic reprogramming.

ATP synthesis was reported to be remarkably downregulated due to mtDNA depletion^21^. The results of Fig. 2B also revealed that the ATP levels were decreased in two cell lines. As the downstream genes of HIF-1α, GLUT1 might be upregulated, which initiated the Warburg effect. Although activation of Warburg effect might present in NARFL knockdown cells, ATP levels were still downregulated due to mitochondrial dysfunction and inhibition of oxidative phosphorylation.

Our results delineated that NARFL deficiency induced downregulation of ND2, ND3, ND5, and ND6 but upregulation of ND1 via HIF-1α–DNMT1 axis. Inhibition of HIF-1α–DNMT1 axis restored the effects of NARFL insufficiency. First, DNMT1 upregulation would translocate into mitochondria and increase the methylation of mitochondrial D-loop, which led to downregulation of ND genes^13^. Second, increased DNMT1 also affected the methylation of nuclear genes, such as TFAM. It was reported that TFAM was a mitochondrial transcription factor encoded by nuclear genes and had different effects on mtDNA heavy and light chains, which regulated the replication and transcription of mtDNA^22–25^. ND6 gene is located on the light chain of mitochondrial genome, but others are on the heavy chain. Methylation of TFAM could promote the expression of ND1 genes and downregulated the transcription of other ND genes^24^. However, this could not account for these different behaviors of ND4 and ND4L expressions and other mechanisms might be involved apart from epigenetic mechanisms.

Drug resistance was measured using Cisplatin and proliferation curves were plotted in two cell lines. NARFL knockdown caused drug resistance via HIF-1α pathway, which wound drive cell survival in lung cancer patients treated with Cisplatin. While cell proliferation did not significantly affect, invasion and migration were increased in NARFL knockdown cells. These results suggested that NARFL deficiency would promote drug resistance and cancer metastasis, and might lead to worse prognosis though DNMT1-mtDNA axis. The results of survival analysis confirmed our hypothesis that patients with NARFL deficiency had a poor survival rate, which could be a predictor for prognosis in lung cancer patients.

Thus, we found that knockdown NARFL affected the mitochondrial functions via HIF-1α–DNMT1 axis, which downregulated the expressions of ND genes and mtDNA copy number. Our results presented that HIF-1α activation induced by NARFL deficiency was responsible for drug resistance and cell migration. Lung cancer patients with NARFL deficiency had a poor survival rate and we established an epigenetic mechanism for lung cancer treatment. These findings might bolster the potential value of NARFL deficiency as a disease marker for treatment and prognosis in lung cancer patients.

### Supplementary Information

Supplementary Figures.

## Data Availability

The datasets used and/or analyzed during the current study available from the corresponding author on reasonable request.
