# Supplementary material for: NARFL deficiency caused mitochondrial dysfunction in lung cancer cells by HIF-1α–DNMT1 axis
Source: Sci Rep. 2023 Oct 11;13:17176. doi: 10.1038/s41598-023-44418-7 (PMC10567771; doi:10.1038/s41598-023-44418-7)

DATA SUPPLEMENT

**NARFL deficiency caused mitochondrial dysfunction in lung cancer cells by HIF-1α-DNMT1 axis**

Hongzhou Liu ^a,b,c,#^, Xueqin Wu ^a,#^, Tianrong Yang ^a,#^, Fei Huang ^a,#^, Ying Xu ^a,^*, Jie Peng ^a,^*

Hongzhou Liu ^a,b,c,#^, Xueqin Wu ^a,#^, Tianrong Yang ^a,#^, Fei Huang ^a,#^, Ying Xu ^a,^*, Jie Peng ^a,^*

a School of Clinical Medicine, the First Affiliated Hospital of Chengdu Medical College, 783# Xindu Avenue, Chengdu, Sichuan Province, 610500, PR China.

b Department of Medical Laboratory, the Central Hospital of Wuhan, Tongji Medical College, Huazhong University of Science and Technology, 26# Shengli Road, Wuhan, Hubei Province, 430014, PR China.

c Department of Clinical Laboratory, the Third People’s Hospital of Chengdu, 82# Qinglong Street, Chengdu, Sichuan Province, 610014, PR China.

# They contributed to this article equally.

* Corresponding author.

Original data

Figure 1


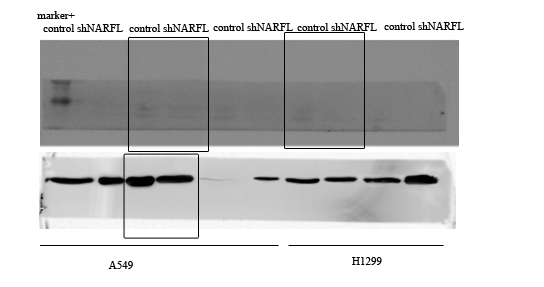


&：The blots in the box appeared in the Figure 1, other sections were not included.








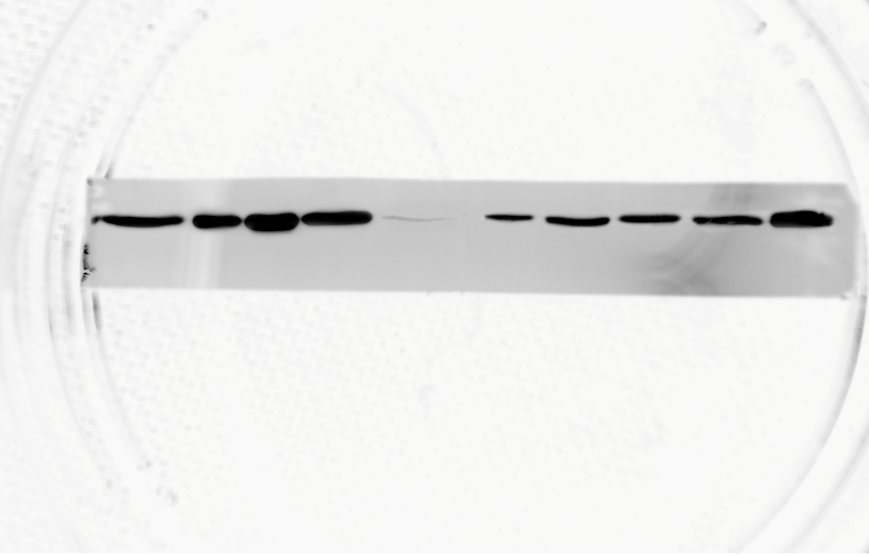


The two figures were original visions.


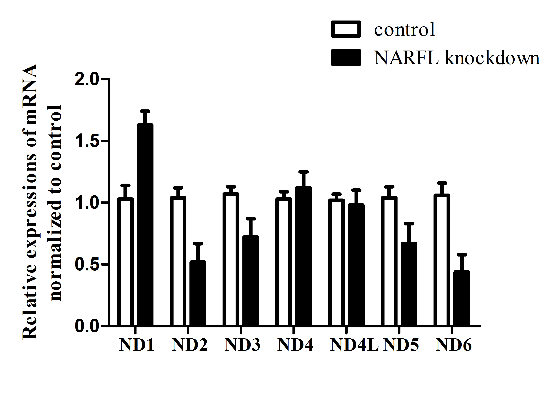


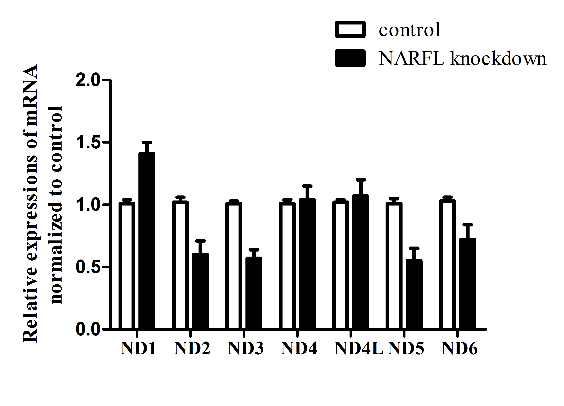


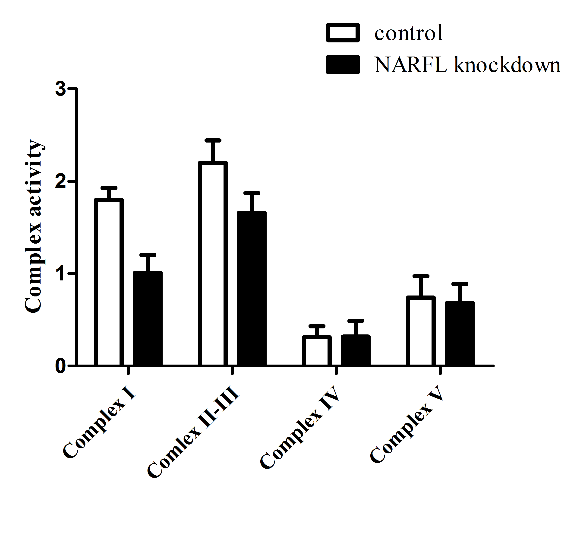


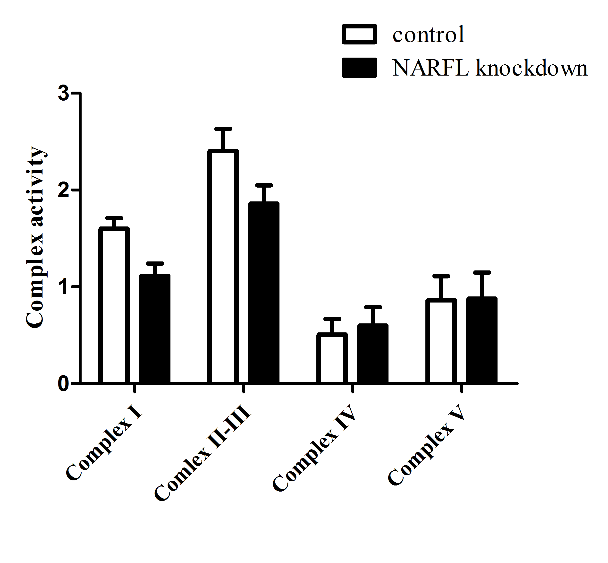


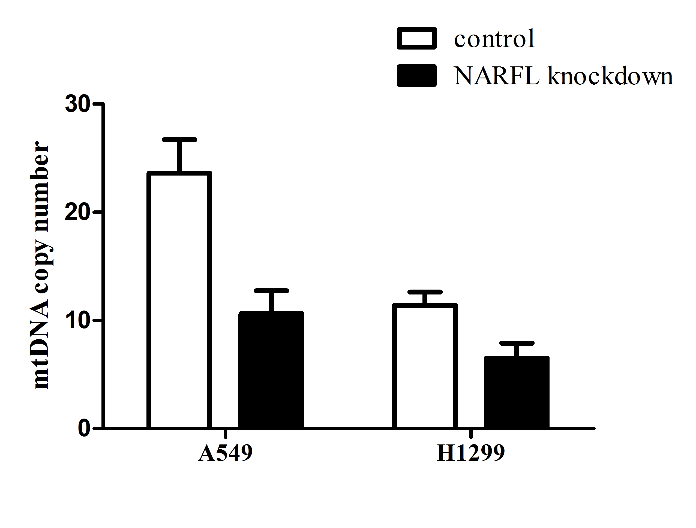


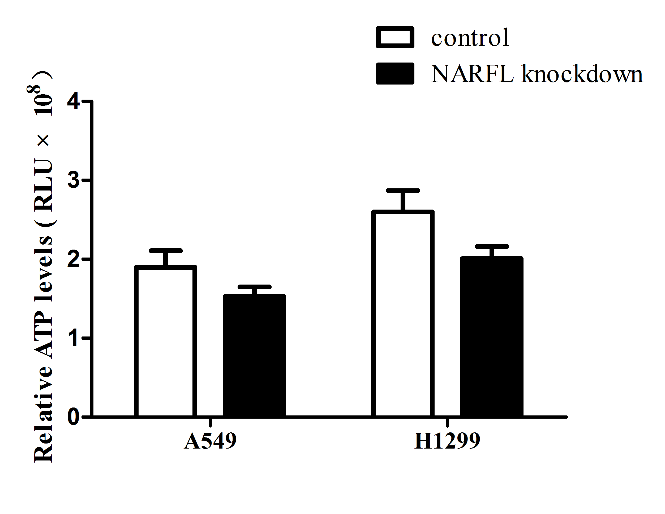


Figure 2


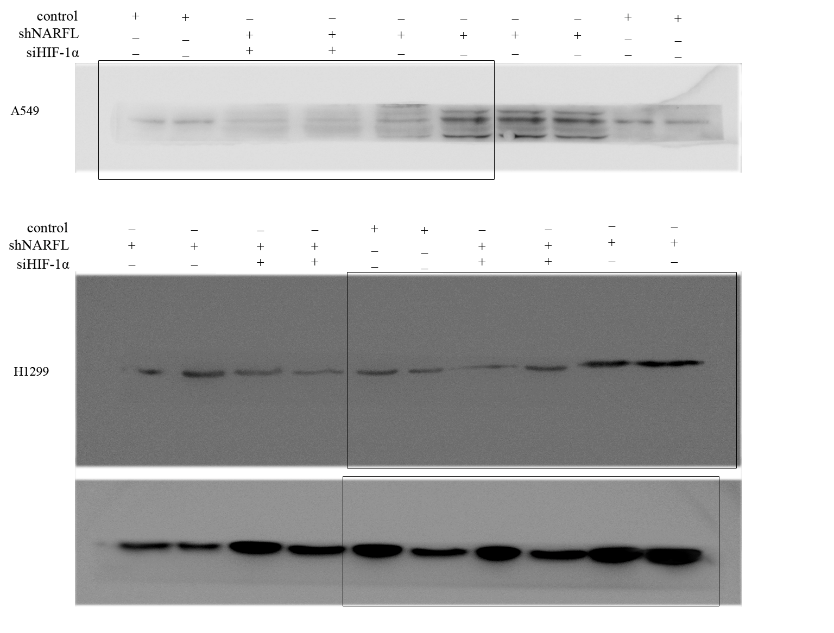


&：The blots in the box appeared in the Figure 2, other sections were not included.













­



The three figures were original visions.


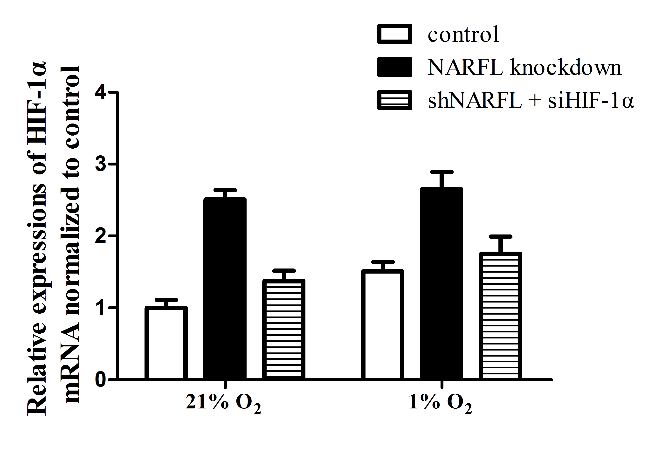


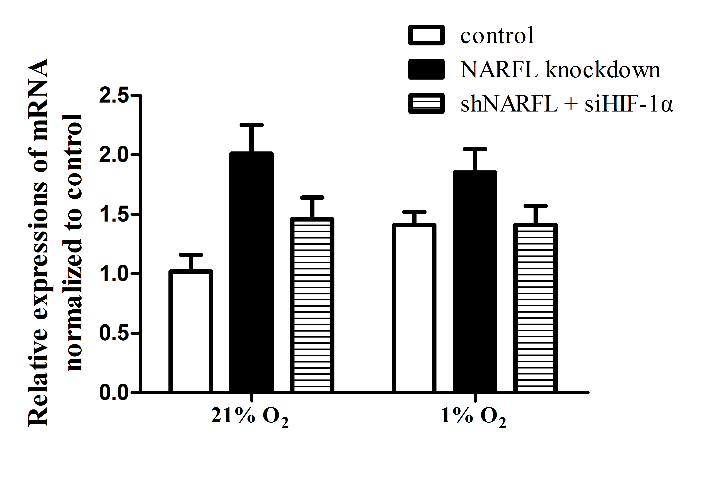


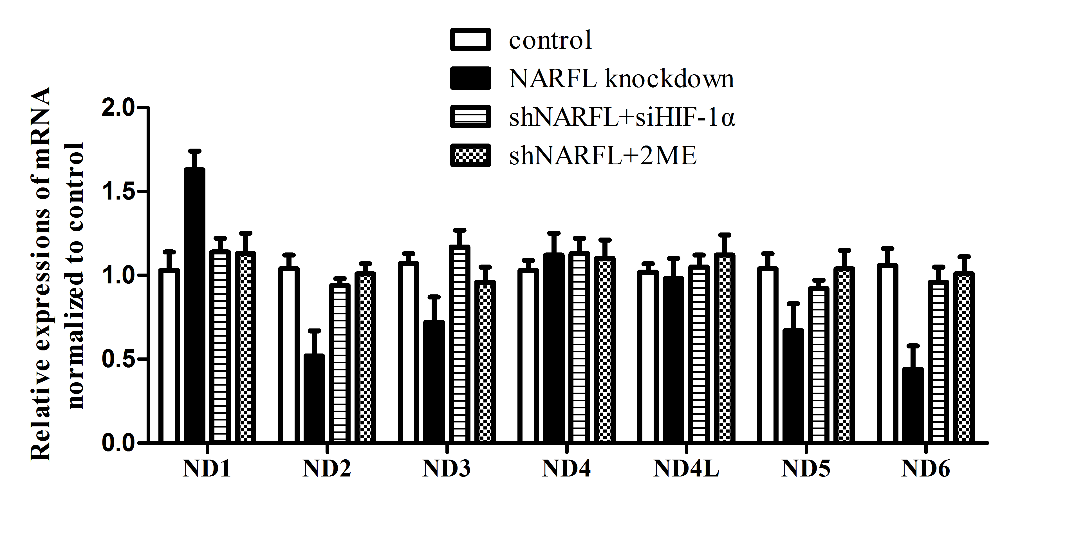


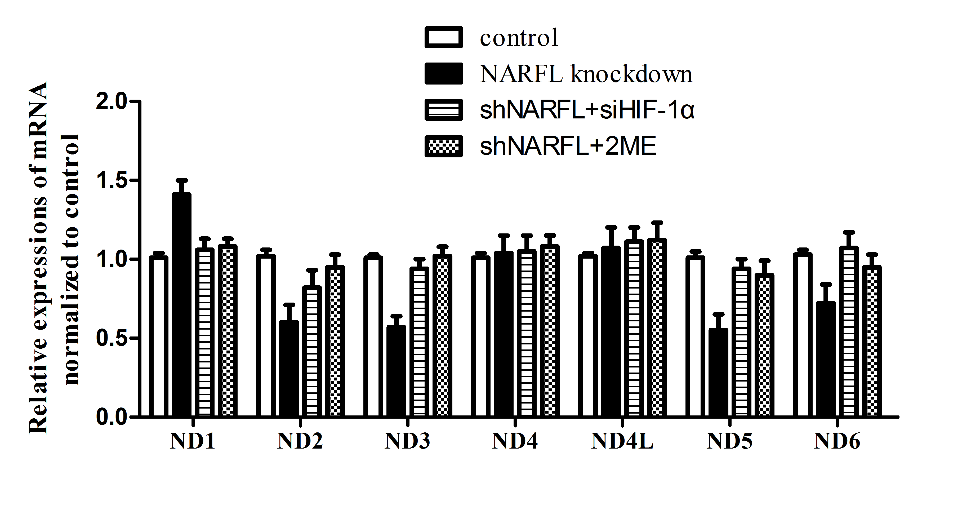


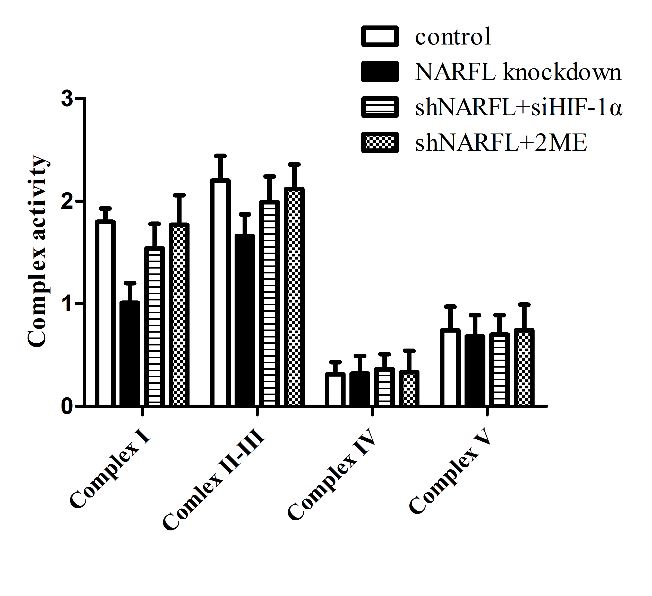


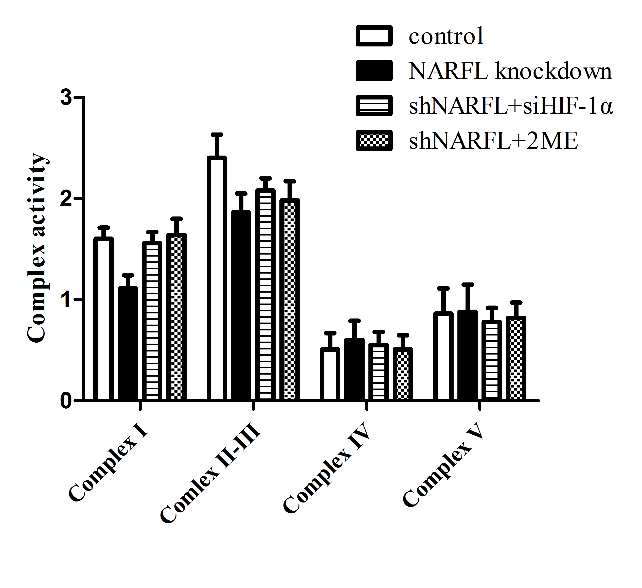


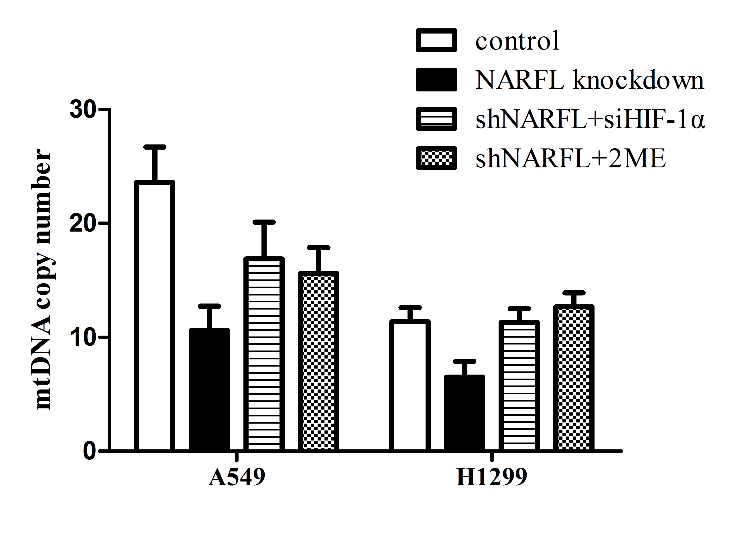


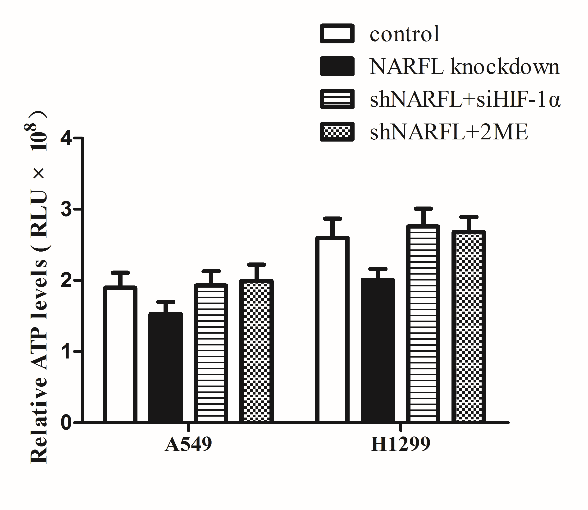


Figure 3


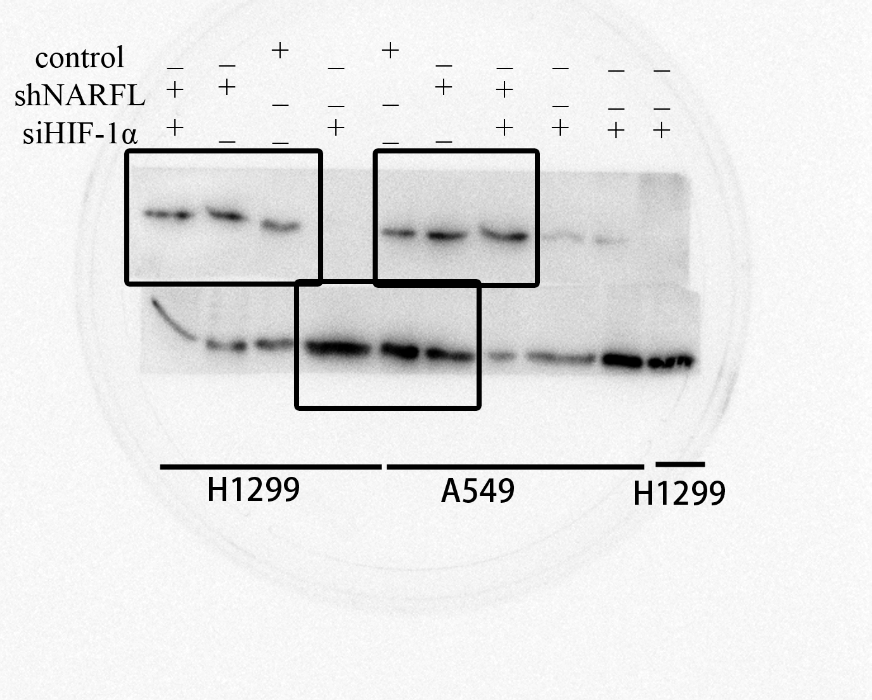


&：The blots in the box appeared in the Figure 3, other sections were not included.


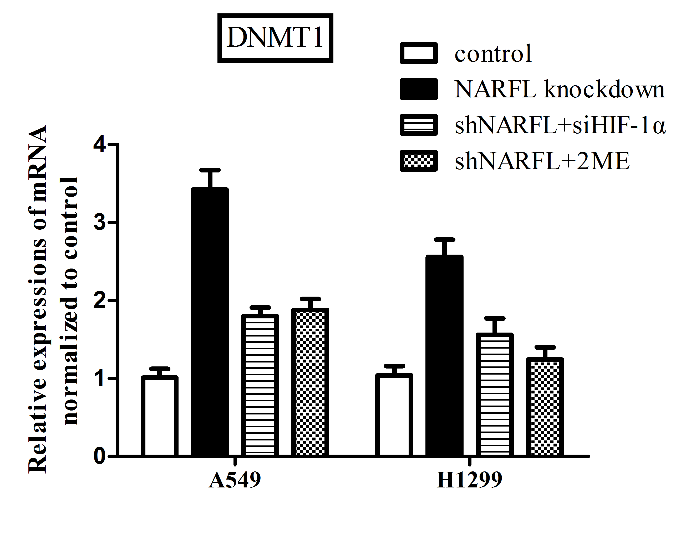


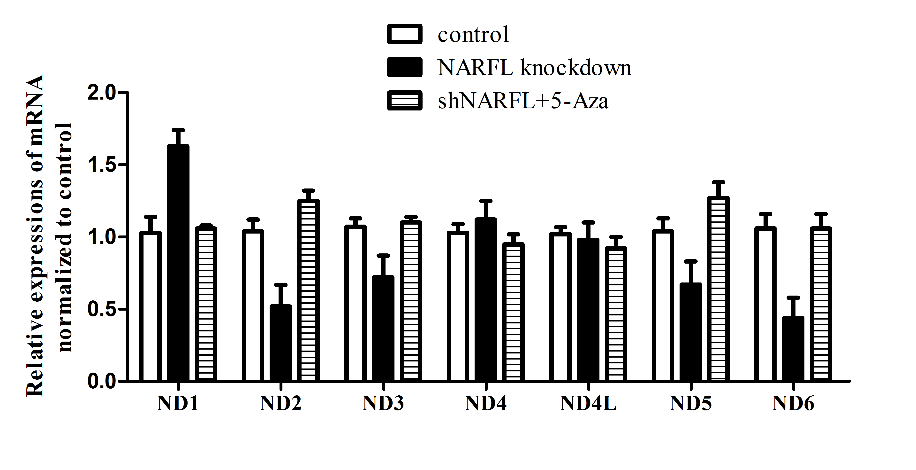

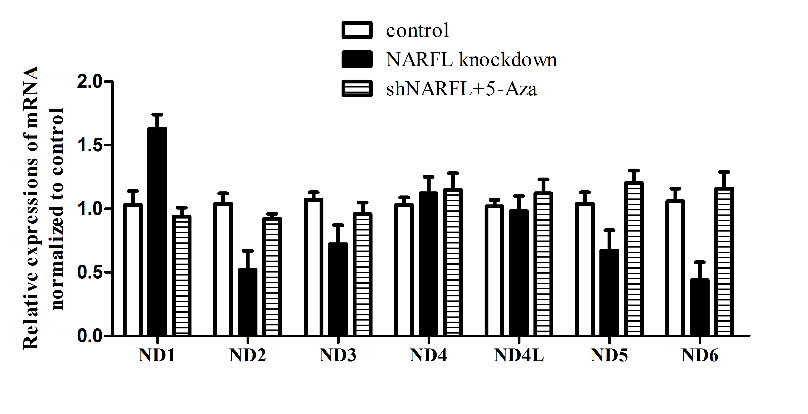


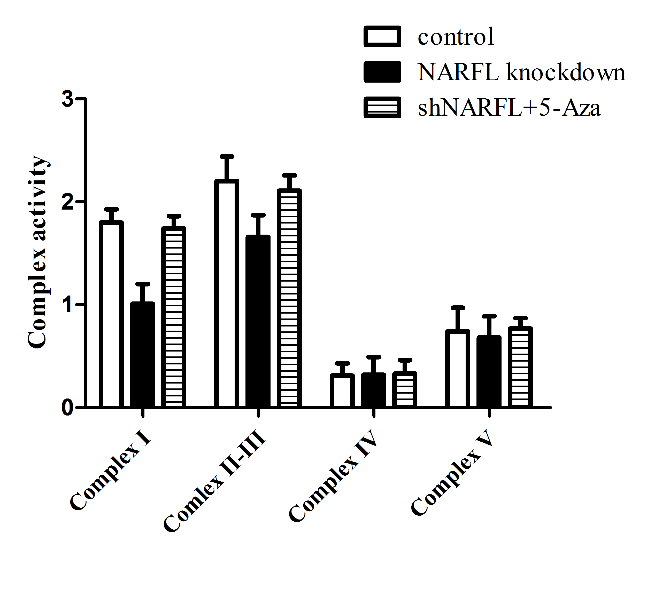


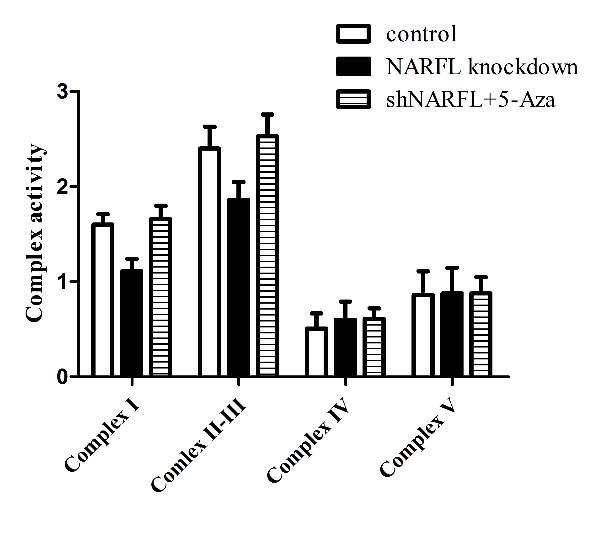


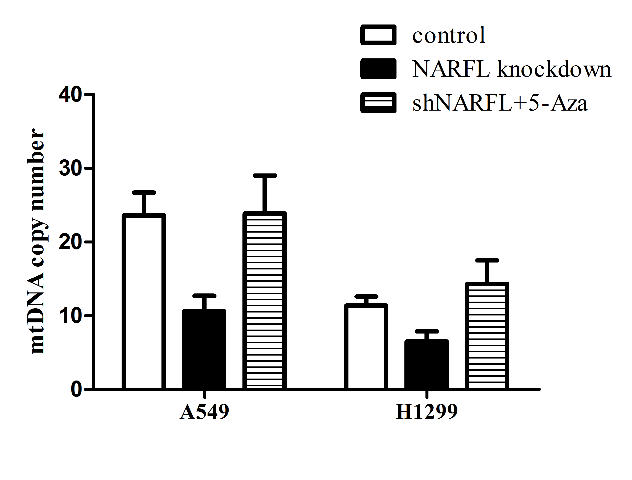


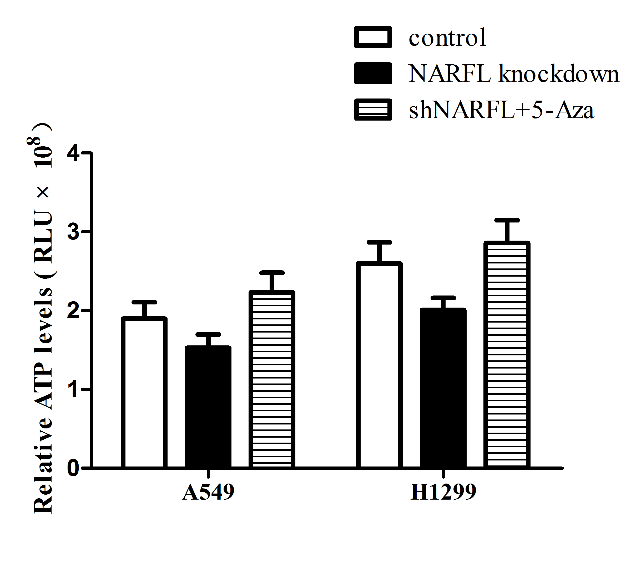


Figure 4


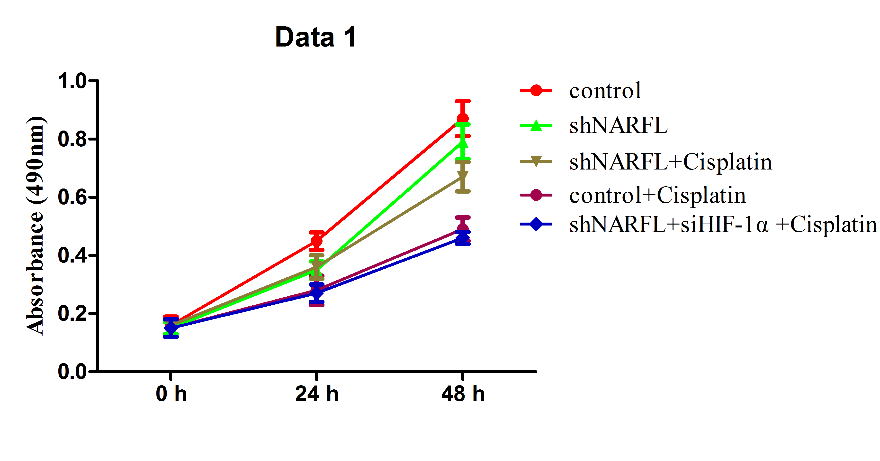


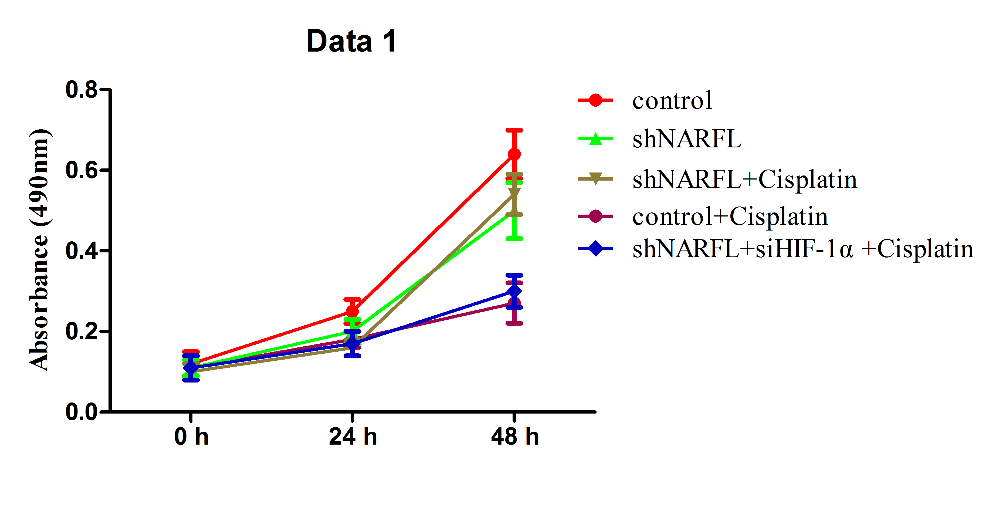


Other results (did not discuss in this manuscript)


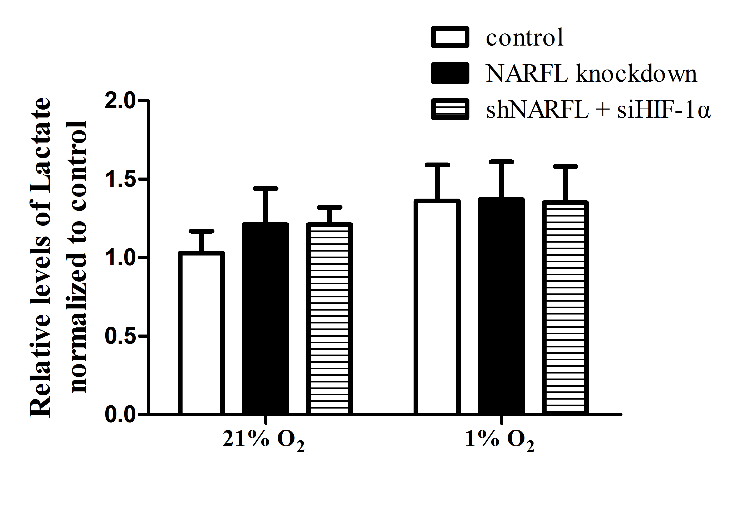


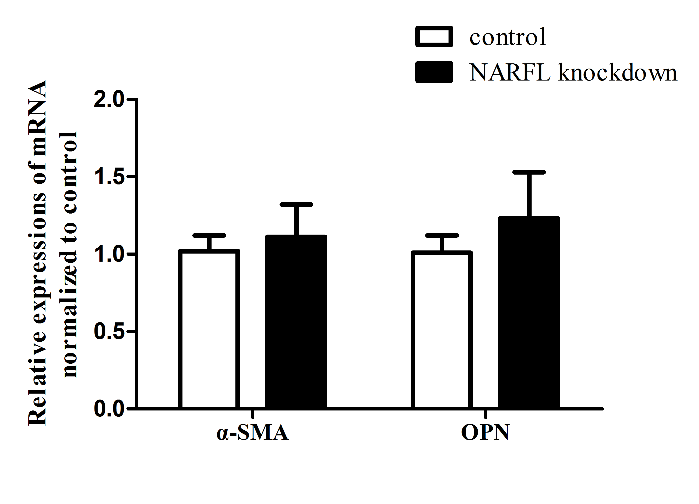


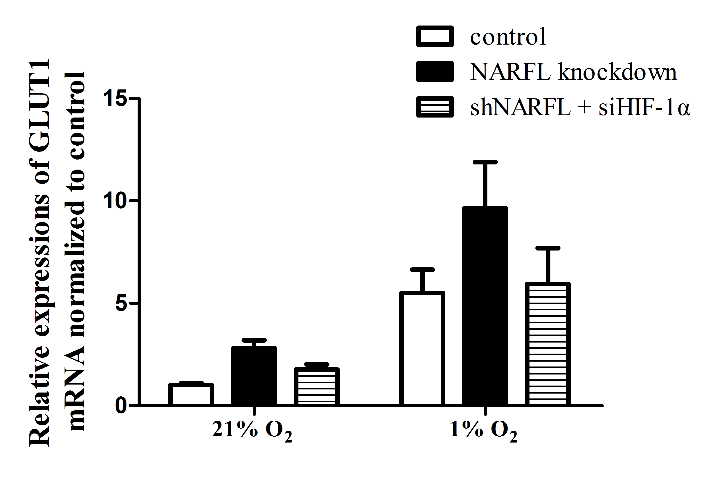


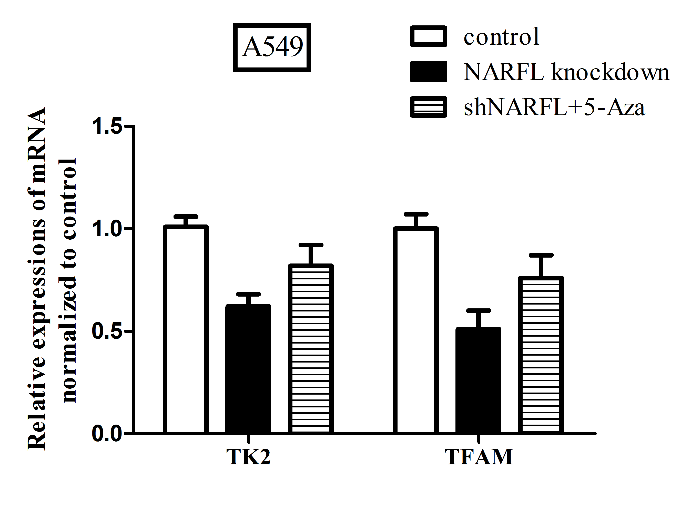


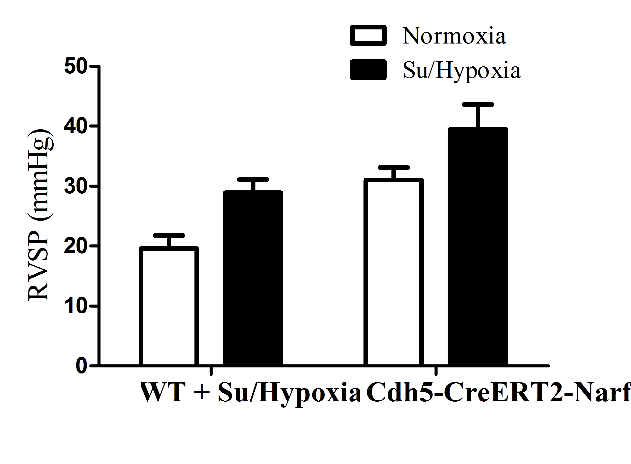


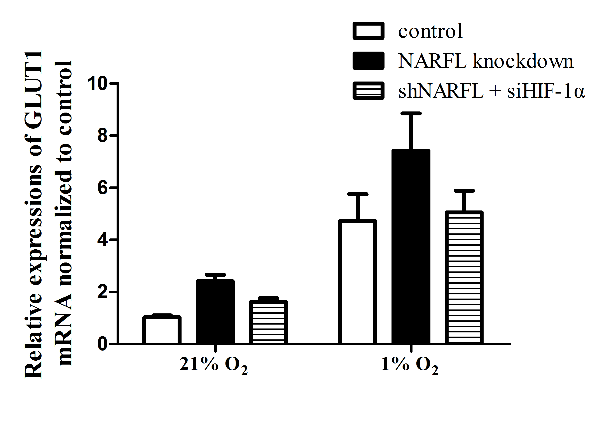


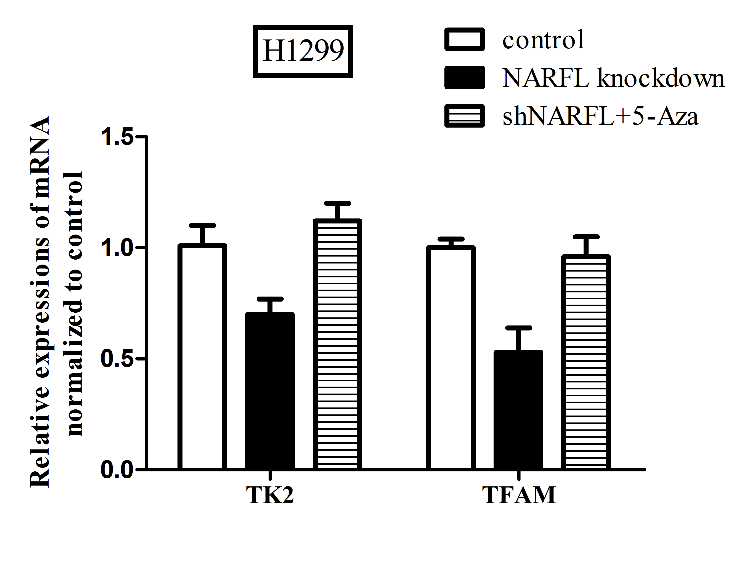


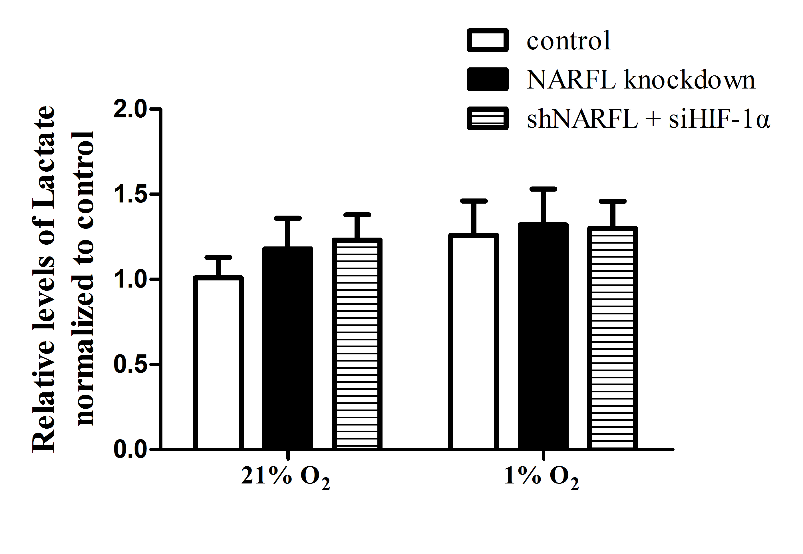


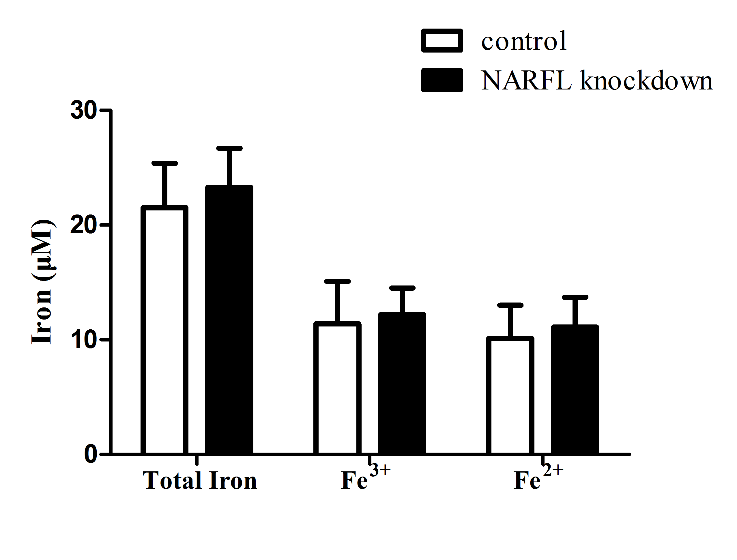


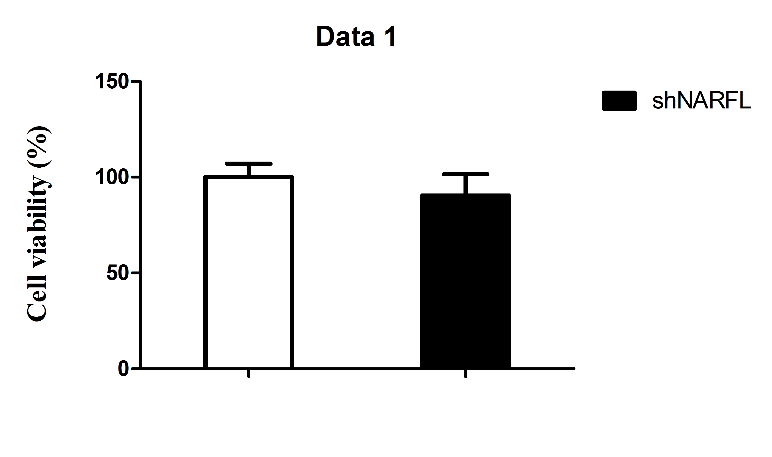

Supplement: Supplementary file 1 — Supplementary Figures. [file 41598_2023_44418_MOESM1_ESM.docx]
